# COALA: A Protocol for the Avoidance and Alleviation of Congestion in Wireless Sensor Networks

**DOI:** 10.3390/s17112502

**Published:** 2017-10-31

**Authors:** Dionisis Kandris, George Tselikis, Eleftherios Anastasiadis, Emmanouil Panaousis, Tasos Dagiuklas

**Affiliations:** 1 Department of Electronic Engineering, Technological Educational Institute of Athens, 12243 Athens, Greece; 2 Department of Computer Systems Engineering, Piraeus University of Applied Sciences, 12241 Athens, Greece; gtselikis@puas.gr; 3 School of Engineering/Computer Science and Informatics, London South Bank University, London SE1 0AA, UK; e.anastasiadis@lsbu.ac.uk (E.A.); tdagiuklas@lsbu.ac.uk (T.D.); 4 Department of Computer Science, University of Surrey, Guildford GU2 7XH, UK; e.panaousis@surrey.ac.uk

**Keywords:** Wireless Sensor Networks, congestion avoidance, congestion control, load balancing, energy efficiency, routing protocol

## Abstract

The occurrence of congestion has an extremely deleterious impact on the performance of Wireless Sensor Networks (WSNs). This article presents a novel protocol, named COALA (*COngestion ALleviation and Avoidance*), which aims to act both proactively, in order to avoid the creation of congestion in WSNs, and reactively, so as to mitigate the diffusion of upcoming congestion through alternative path routing. Its operation is based on the utilization of an accumulative cost function, which considers both static and dynamic metrics in order to send data through the paths that are less probable to be congested. COALA is validated through simulation tests, which exhibit its ability to achieve remarkable reduction of loss ratios, transmission delays and energy dissipation. Moreover, the appropriate adjustment of the weighting of the accumulative cost function enables the algorithm to adapt to the performance criteria of individual case scenarios.

## 1. Introduction

The operation of a WSN is interdependently correlated with the existence of data traffic. Network congestion is one of the most serious problems encountered in the management of the data traffic within a WSN. Congestion occurs when current traffic load exceeds available transmission ability at any point in the network. Congestion has an absolutely detrimental impact on WSN performance [1]. More specifically, congestion procures the overflow of node buffers, the degradation of the overall channel quality, and the increase of both loss rates and transmission delays.

This article proposes a novel protocol that aims, by ruling the routing process, not only to prevent the occurrence of congestion, but also to deter the dispersion of oncoming congestion in WSNs. This protocol performs the discovery of the routing paths that are less likely to be congested, based on the computation of a suitably formulated cost function. This cost function takes into consideration a collection of static and dynamic metrics that are related with congestion.

The remainder of this article is organized as follows. Section 2 outlines various existing protocols for congestion avoidance and in congestion control WSNs. The description of the proposed congestion protocol takes place in Section 3. In Section 4, the performance evaluation of the proposed protocol is performed through the description and analysis of simulation results. Finally, Section 5 concludes the article.

## 2. Related Work

Congestion is a phenomenon that comes along either interference in the communication medium, which is caused by the concurrent transmission of many nodes, or buffer overflow, which is caused by the fact that incoming traffic load in a node exceeds its buffer capacity. The confrontation of congestion is the subject of numerous scientific research works [1,2,3,4,5,6,7]. Some of them, introduce *congestion avoidance protocols*, which propose proactive tasks in order to prevent congestion occurrence. These protocols normally involve MAC and network layer operations. Some other research works propose *congestion control protocols* that act reactively to the existence of congestion in order to mitigate it. Protocols of this kind normally involve MAC and network layer operations, and in some cases they also use transport layer actions. Additionally, cross layer interaction between transport and underlying layers is an efficient way of congestion control while MAC layer provides channel status that can be incorporated in congestion control mechanisms [1].

The detection of congestion can be performed by taking into consideration one or a combination of specific performance metrics. The most popular of them are: packet loss, buffer occupancy, delay, packet service time and packet inter-arrival time [2,3].

The notification of congestion occurrence can be either *explicit*, where relative informing messages are sent by congested nodes to other nodes, or *implicit*, where the notifying information is incorporated in data packet headers or in ACK packets that are piggybacked. Explicit notification is unfavorable because it adds substantial traffic load to the already jammed network [4,5].

The mitigation of congestion can be pursued by either traffic control or resource control or a combination of them. When *traffic control* is applied, the quantity of the packets injected into the network is suitably decreased in order to alleviate both traffic load and congestion. However, traffic control is not efficient in event-based applications where any restriction in the transmission of data is inacceptable. When *resource control* is applied, data packets are routed through alternative paths that are not congested. Yet, in this way extra delays or even routing loops may be caused [6,7].

CODA, proposed by Wan et al. [8], is one of the most well -known protocols, which aims at the achievement of congestion avoidance and control. Its operation is based on flow control. It introduces the idea of a control mechanism by which every node that detects the occurrence of congestion, sends backpressure messages to its data source nodes. Every source node that receives backpressure signals either throttles its sending rates or drops packets based on the local congestion policy adopted. Additionally, source nodes start throttling their sending rates as soon as they do not receive, at predefined time, feedback messages sent to them by sink.

Ahmad and Turgut [9] proposed an alternative path routing protocol which uses the ratio of the numbers of downstream to upstream nodes along with the queue sizes of the downstream nodes in order to detect congestion and reallocate traffic through alternate routes.

PACA, proposed by Kandris et al. [10], pursuits congestion avoidance by circumventing nodes that are either located close to the sink, or have more downstream than upstream neighbors or have very frequent data transmissions.

In [11] Sergiou et al. introduce two very promising lightweight schemes for congestion avoidance and control. In the first of them, named DAlPaS Hard, data flows are forced to change their path in order not to congest the receiving node based on a multivariable utility function. In the second of them, named DAlPaS, each node attempts to serve just one flow and if this is unavoidable the DAlPaS Hard algorithm is executed.

Huang et al. proposed TALONet [12], which uses both traffic control and resource control to avoid congestion. Specifically, TALONet combines various levels of transmission power in order to relieve existing congestion in data link layer, along with buffer management to avoid congestion in buffer level, and multi-path routing in order to relay congested traffic flows through alternative paths.

HTAP [13], proposed by Sergiou et al., is a scalable protocol aiming to minimize congestion and assure reliable data transmissions in event-based networks through resource control. As soon as congestion occurrence is detected, alternative paths are created and nodes are hierarchically levelled in these paths and exhausted nodes are bypassed in order to achieve load balancing.

He et al. proposed TADR [14] which uses shortest paths in order to route data when there is no congestion. As soon as congestion is detected, the concept of potential fields is used, by taking into consideration network depth and normalized queue length, in order to distribute data through multiple paths consisting of idle and under-loaded nodes so as to circumvent congested areas.

Tao and Yu introduced ECODA [15], where packets are dynamically prioritized, using their initial static packet priority, hop-count and the time from the packet generation to current time. ECODA proposes buffer related metrics so as to detect congestion. The congestion status is piggybacked in packets. When receiving such a back-pressure message, the source node either reduces its transmission rate, or accordingly adjusts the rate for different paths if multiple paths exist.

Kang et al. proposed the TARA protocol [16]. In TARA, as soon as emerging congestion is detected in a node, by measuring both the buffer occupancy and the channel load, this node is considered to be a hot-spot node. Next, traffic is deflected from the hot-spot node through a so-called distributor node along a detour path and reaches the so-called merge node, where the flows are merged. As soon as congestion has been alleviated the network stops using the detour path.

Jan et al. introduced PASCCC [17], which is an energy-efficient application specific clustering congestion control protocol. In PASCCC, data packets are prioritized as high priority and low priority packets according to the type of their content. During congestion low priority packets are discarded.

Table 1 enlists in comparison basic characteristics of both the aforementioned protocols and the proposed in this research work protocol.

## 3. Proposed Protocol Description

Following the above-mentioned introduction to well-known protocols for the avoidance and or control of congestion in WSNs, COALA (*COngestion ALleviation and Avoidance*), which is the proposed in this article protocol of this kind, is introduced in this section.

COALA protocol, operating proactively, uses simple static information of network nodes in order to perform data routing through the paths that are less probable to be congested. In the face of imminent congestion, COALA acting reactively, uses implicit notification of congestion and applies resource control with the intention of preventing the further diffusion of congestion.

### 3.1. Preliminary Considerations and Terms

**Figure 1:**
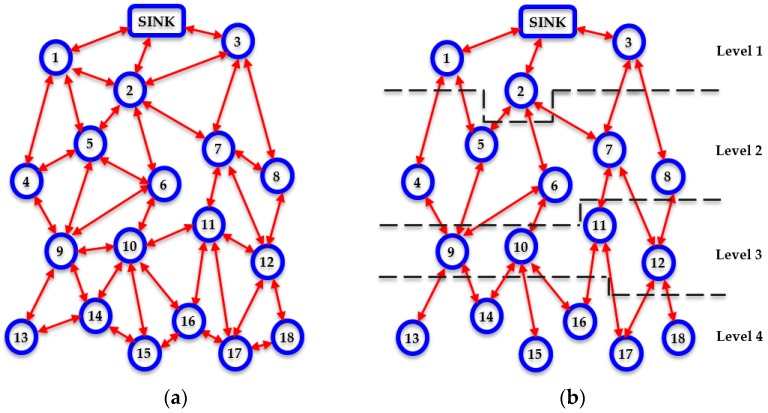
A typical example of a WSN topology in: (**a**) Initial arrangement of interconnected network nodes; (**b**) Level-based taxonomy of network nodes.

First, a densely deployed WSN where homogeneous nodes are positioned in a uniformly random way, such as the one illustrated in Figure 1a, is considered to be the reference point for the protocol description. It is assumed that there are is one sink. It is also supposed that every individual node knows both its location and the location of the sink.

Additionally, it is assumed that incidents referred here as *events*, are created and all nodes that are located within the range of each event, send related data to the sink. Thus, the number of data sources is variable. Also, all nodes are considered to have the same transmission and sensing range. Moreover, it is supposed that multihop routing through direct neighboring nodes is utilized, whenever the sink is not placed within the transmission range of the node that relays data to the sink. Furthermore, CSMA is considered to be used as the medium access control (MAC) protocol.

For every individual node *i*:

- the area where the signals sent by node *i* can reach, is defined as *Transmission Range TR*(*i*)
- the minimum number of hops for node *i* in order to reach the sink, is defined as *level L*(*i*)
- every node *j* that is placed within *tr*(*i*) and has *L*(*j*) = *L*(*i*) ± 1, is defined as *Neighboring Node NN*(*i*)
- the ratio of the total number of *NN*(*i*) whose level *L* is greater than *L*(*i*) to the total number of *NN*(*i*), is defined as *Vicinity Index VI*(*i*)
- the ratio of the accumulative participation of a node in data flows over time is referred as *Popularity Index PI*(*i*)
- the so called *Availability Index AI*(*i*) expresses either the ability (when having value 1) of the node to relay data or the unavailability (when having value 0) of the node to do so, because the node has either limited buffer space, or limited energy or because its neighboring nodes that have lesser level are blocked

### 3.2. Initialization Phase

The initialization phase of COALA aims to perform all the initial calculations of both the level *L* and the neighbor table of all networks nodes. These calculations are necessary for the inception of the congestion avoidance algorithm and this is why it is executed only once.

Specifically, this phase is initiated as soon as the sink transmits a “hello” message, which includes the sink ID number along with its level *L*, which is equal to 0. Next, every node that receives this message, sends back to the sink a corresponding acknowledgement message that includes its ID number. As soon as the sink receives such an acknowledgement message from a node, it sends another message back to this node, which confirms that this node is a neighboring node of the sink and that it is a level 1 node. Every level 1 node initiates its neighbor table, which includes the details of the sink and transmits a “hello” message, which includes its ID number, its level *L*, which is equal to 1, its position, its current energy, and its current buffer occupancy. Every node that receives this message, sends back to the transmitting node a corresponding acknowledgement message that includes the current values of its neighbor table parameters, which are explained later on. As soon as the level 1 node receives such an acknowledgement message from a node, it sends another message back to this node that includes its updated neighbor table along with a confirmation that this node is its neighboring node. If the new neighboring node has not already acquired a level number, then it is recognized as a next (i.e., 2) level node.

This procedure carries on until every individual node in the network not only has been assigned a corresponding level number, as illustrated in Figure 1b, but also has become aware of its neighbor table. In this way, each node constructs an overall view of the network topology and becomes aware of all the available routing paths towards the sink, avoiding the formation of routing loops.

### 3.3. Steady-State Phase

The steady-state, which is explained in this subsection, begins as soon as the initialization phase is completed. Its operation is mainly based on the utilization of the neighbor tables that, as mentioned above, have been created, during the initialization phase, for all network nodes. A typical neighbor table of a network node contains the current values of the following parameters:

- node ID,
- level number
- position
- energy
- buffer occupancy
- popularity index
- availability index
- vicinity index

The overall data routing process, during the steady-state, is based on the current values of all neighbor tables that are dynamically updated. Specifically, similarly to [11] every network node, which has data to send to the sink, examines its own neighbor table and discovers the candidate recipients among its neighbors who have smaller level number, i.e., its neighbors, which are located at a level closer to the sink, if any.

Therefore, at this point, each node neglects all of its connections with other nodes, which are located either at the same level with itself or at lower levels and focuses at the nodes that are placed at an upper level in the network taxonomy, as shown in Figure 1b.

In the case that there are at least two candidate recipient nodes having *AI* = 1, COALA protocol suggests that the transmitting node must relay its data through the node that has the minimum vicinity index. For instance, referring to Figure 1b, if node 14 has data to send upwards to the sink, then it has two candidate nodes through which it can relay its data, i.e., node 9 and node 10. If these nodes are both available (i.e., have *AI* = 1) then node 9 will be selected, since *VI*(9) = 2/5 = 0.4 and *VI*(10) = 3/4 = 0.75. This is because node 9 may receive data from 2 lower level nodes and transmit data to 3 upper level nodes, while node 10 may receive data from 3 lower level nodes and transmit data to 1 upper level node. Thus, theoretically node 10 is more likely to be congested than node 9 if normalized flow rate patterns are considered. This initial routing criterion enhances the corresponding initial consideration suggested in [11].

This procedure is carried on until the whole routing path has been determined. The selected path information is stored in the header of the data packets transmitted. In this way, every time that a node has data to send to the sink, a dynamically updated spanning tree is created in order to route all data load through the theoretically less probable to be congested shortest path. It is important to notice that this process is based on purely static information, which is well-known as soon as the steady-phase is terminated.

However, in the case that the node that is selected to be the next to receive data, becomes unavailable (due to either buffer or energy limitations or lack of available upper level neighbors) the routing process necessarily changes.

Specifically, the data has to be relayed via an alternative path. For this reason, COALA protocol proposes the utilization of a multivariable cost function that aims to evaluate the overall cost of every neighboring node, by taking into consideration the buffer occupancy, the dissipated energy, the level number, the availability index, the popularity index, the vicinity index and the geographical distance of this node. The mathematical representation of this cost function is defined in (1):

(1) $$AC\left(i\right)=\left[w_{\tilde{BO}}\cdot \tilde{BO}\left(i\right)+w_{\tilde{DE}}\cdot \tilde{DE}\left(i\right)+w_{\tilde{L}}\cdot \tilde{L}\left(i\right)+w_{\tilde{VI}}\cdot \tilde{VI}\left(i\right)+w_{\tilde{PI}}\cdot \tilde{PI}\left(i\right)+w_{\tilde{D}}\cdot \tilde{D}\left(i\right)\right]\cdot AI\left(i\right)$$

where:

AC(i) denotes the accumulative cost of node *i*
  $\tilde{BO}\left(i\right)$ denotes the normalized buffer occupancy of node *i*
$\tilde{DE}\left(i\right)$ denotes the normalized dissipated energy of node *i*
$\tilde{L}\left(i\right)$ denotes the normalized level number of node *i*
$\tilde{VI}\left(i\right)$ denotes the normalized vicinity index of node *i*
$\tilde{PI}\left(i\right)$ denotes the normalized popularity index of node *i*
$\tilde{D}\left(i\right)$ denotes the normalized geographical distance of node *i* from the transmitting node
AI(i) denotes the availability index of node *i*
$w_{\tilde{BO}},\;w_{\tilde{DE}},w_{\tilde{L}},w_{\tilde{VI}},w_{\tilde{PI}},w_{\tilde{D}}$ are the corresponding weighting factors

Once the accumulative cost has been calculated for all alternative neighboring nodes, COALA routing algorithm selects the node that has the minimum value of accumulative cost as the next recipient of the data in the routing path towards the sink.

The buffer occupancy metric is considered in order to prefer nodes that have more free space in their buffers. The dissipated energy parameter is used so as to avoid the use of nodes that have low energy reserves. The level number is considered in order to give priority to upper level nodes, so that data are routed towards the sink. The vicinity index metric is used in order to avoid the utilization of nodes that have plenty of probable data suppliers and few data recipients. The popularity index is taken into consideration so as to slide over network nodes that tend to be repetitively busy. For instance, nodes that are located either in centric routing paths or within areas with frequent creation of events have high popularity and thus are more probable to get congested. The geographical distance between neighboring nodes is taken under consideration in order to give increased priority to the data relaying via the closest nodes than the more distant ones. The overall sum of products is multiplied by the availability index in order to prevent the utilization of nodes that are unavailable due to either insufficient energy, or inadequate buffer space or even inaccessible upper level neighboring nodes.

**Figure 2:**
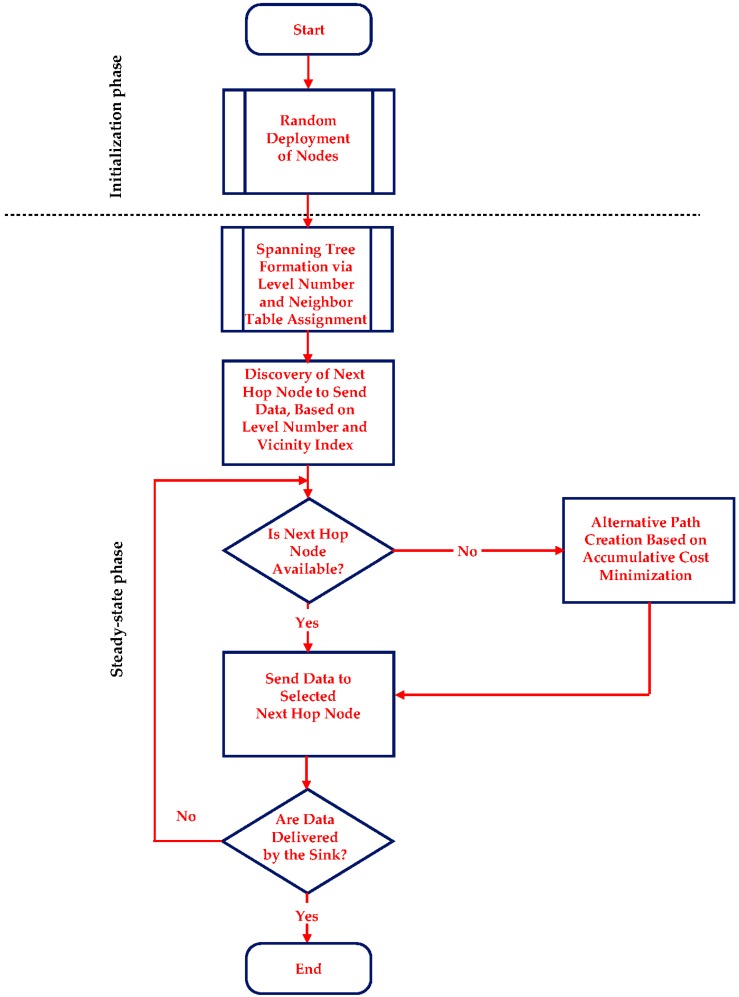
Flowchart of the algorithm of COALA protocol.

Additionally, the existence of the weighting factors in this cost function, aims to support the appropriate adaptation of the algorithm in order to satisfy the different demands of every individual application. For instance, wherever the conservation of energy has major priority, $w_{\tilde{DE}}$ is assigned a greater value. In Figure 2, a descriptive flowchart of COALA protocol is illustrated.

## 4. Proposed Protocol Evaluation

The proposed congestion avoidance protocol has been validated through the utilization of an appropriately developed, simulation environment. The customized console-based simulation platform was built by using C++ programming language according to the methodologies described in [18,19].

Specifically, multiple runs have been executed in order to investigate the protocol performance in comparison with DAlPaS Hard scheme in various scenarios concerning randomly deployed topologies.

### 4.1. Simulation Process

The simulation environment creates a number of user defined nodes, randomly positioned in a user defined geographical area of square shape. The node that is created first plays the role of the unique sink. The user defines the minimum node transmission range that enables two nodes have direct communication and thus be considered as neighboring nodes. The software environment assures that for every individual network node there is at least one routing path from this node towards the sink.

In every single simulation test, several different topologies are created. For every topology, a number of simulation runs is applied. During simulation tests, events are created in arbitrary positions and random time instances and each event range, i.e., a cyclical area that surrounds the event position, was supposed to be varying. Each node, located within an event range, is supposed to sense the occurrence of this event and has to transmit corresponding event notifications to the sink. Since multiple nodes may serve the same event, the sink may receive multiple notifications for the same event. Each event notification is accompanied by a corresponding event message header. This event header contains a time stamp that denotes the time of its generation.

As the event range increases from an initial value to a maximum value, the number of the nodes that are located within the area where the specific event takes place increases too. Subsequently the number of the notification data sent to the sink also arises.

The initial energy of every individual node is considered to range between a high and a low limit value, which are user defined. This condition has been set in order to conform to the fact in real WSNs applications the network nodes have different energy reserves. Additionally, the dissimilarity in the energy levels of the sensor nodes allows simulation tests to demonstrate better the ability of COALA protocol to achieve energy efficient performance.

The configuration parameters along with their values are summarized in Table 2.

### 4.2. Presentation and Appraisal of Simulation Results

**Figure 3:**
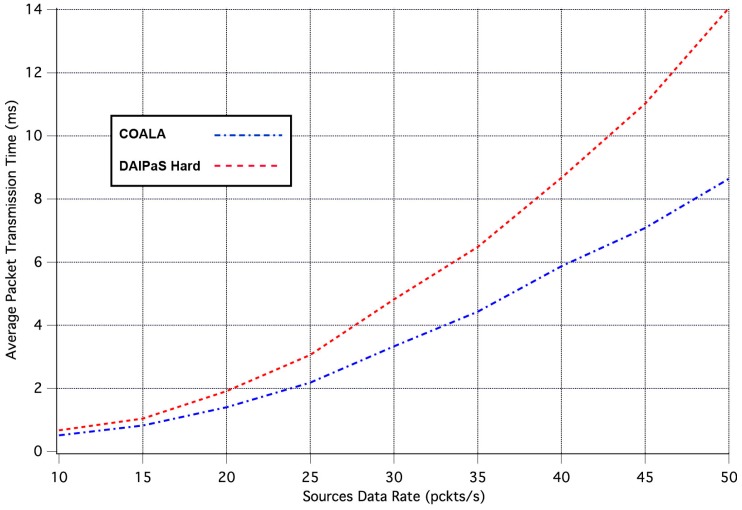
Average Packet Transmission Time vs. Traffic Load Rate.

The first set of simulation tests performed, evaluate how the increase in the rate of data transmission influences the average time it takes for an event notification, sent by a network node that senses the specific event, to reach the sink. The corresponding simulation results are illustrated in Figure 3. In these tests, all weighting factors are considered to be equal to 1.

As it can be seen in Figure 3, in COALA protocol the average packet transmission time not only is less than that in DAlPaS Hard, but it is also more robust against the gradual growth of data transmission rate.

**Figure 4:**
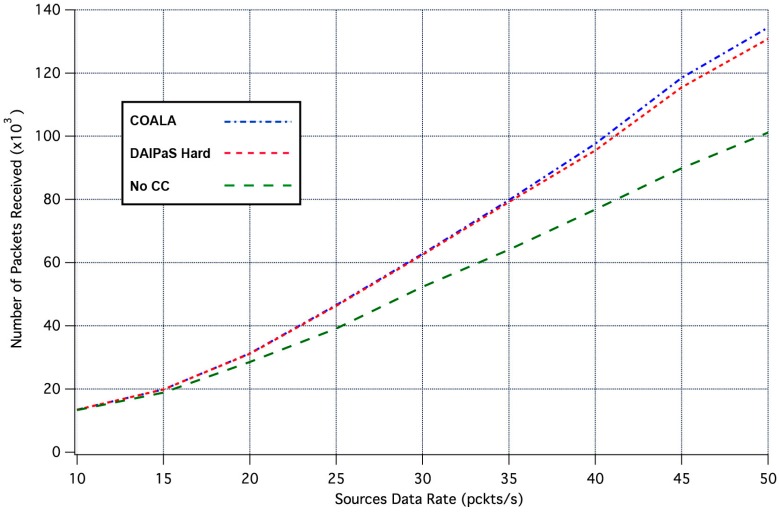
Number of Packets Received vs. Traffic Load Rate.

**Figure 5:**
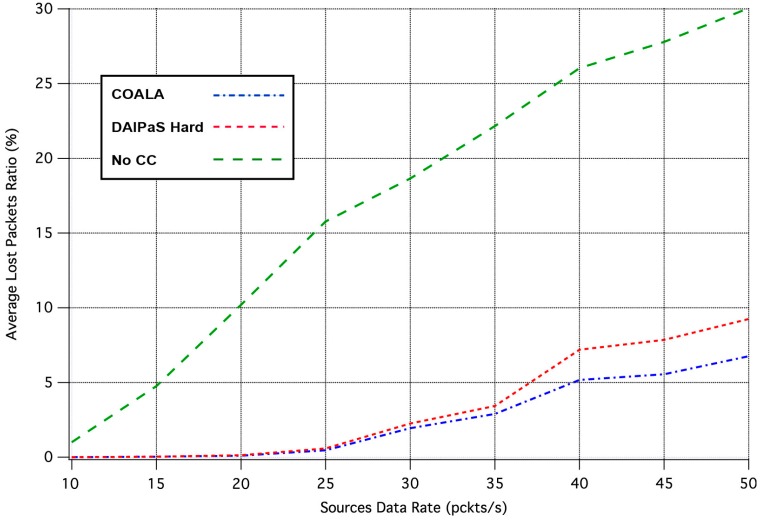
Average Lost Packets Ratio vs. Traffic Load Rate.

Next, the performance of COALA protocol is investigated relatively with how the data transmission is affected by the progressive rise of the of data traffic. This correlation is depicted in Figure 4 and Figure 5, through the graphical representation of the overall number of the data packets received and the percentage of the data packets lost respectively.

The examination of both Figure 4 and Figure 5 makes evident that COALA protocol not only achieves lower data losses but also resists more against the progressive increase of the traffic load.

**Figure 6:**
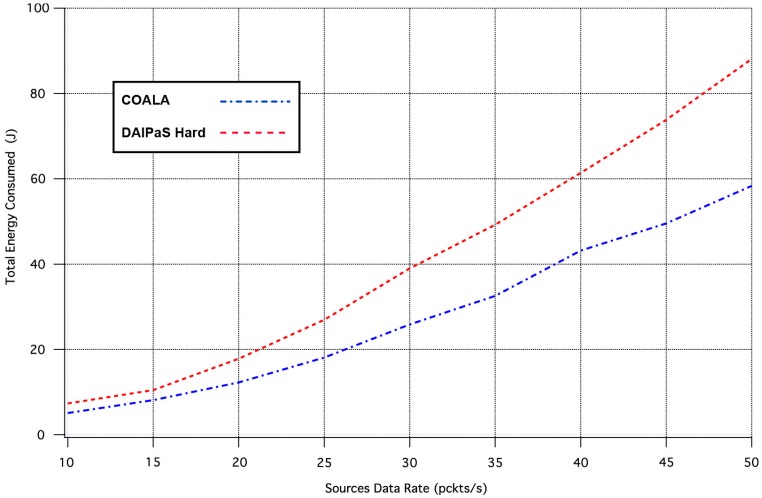
Network Energy Dissipation vs. Traffic Load Rate.

The next performance metric evaluated is the overall energy consumption of the network. Figure 6, illustrates in what way the rise of traffic load increases the network energy dissipation.

The examination of Figure 6 demonstrates that COALA protocol outperforms DAlPaS Hard in energy efficiency increasingly as the traffic load raises.

Finally, a set of simulation tests were performed in order to examine how the variation of the weighting factors of the accumulated cost of nodes deviates the results of COALA protocol utilization. As an example, the way by which the variation of two weighting factors, i.e., $\;w_{\tilde{D}}$ and $\;w_{\tilde{BO}}$ influences the average packet transmission time and the number of lost packages was investigated.

**Figure 7:**
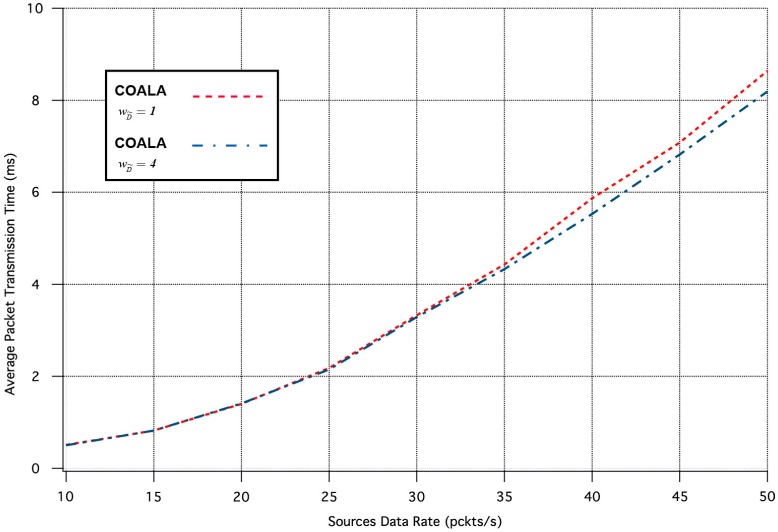
Average Packet Transmission Time vs. Traffic Load Rate for variable $\;w_{\tilde{D}}$.

**Figure 8:**
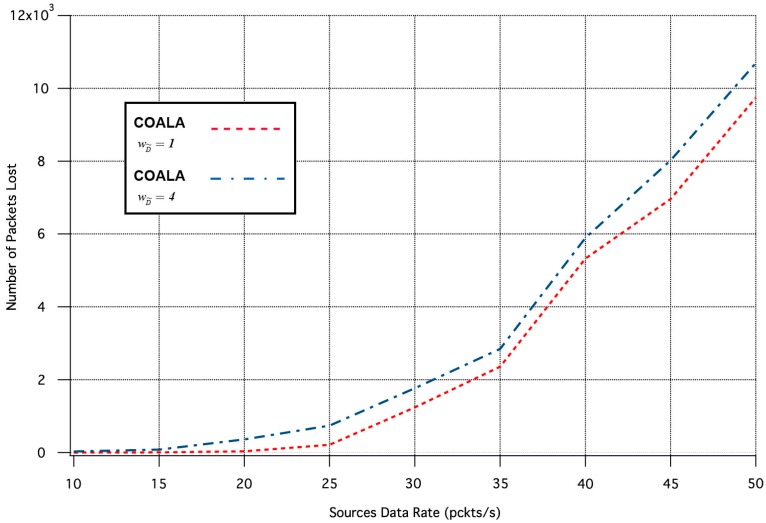
Number of Packets Lost vs. Traffic Load Rate for variable $\;w_{\tilde{D}}$.

Specifically, the influence of the $\;w_{\tilde{D}}$ variation in the two aforementioned metrics is depicted in Figure 7 and Figure 8 correspondingly.

The increase of $\;w_{\tilde{D}}\;$ makes the distance criterion have key priority within the accumulated cost function. The examination of Figure 7 and Figure 8 validates that this increase accelerates the data transmission but deteriorates its quality. This is because data are preferred to be relayed through the closest neighboring nodes although these nodes may have more traffic load than other more distant neighbors.

**Figure 9:**
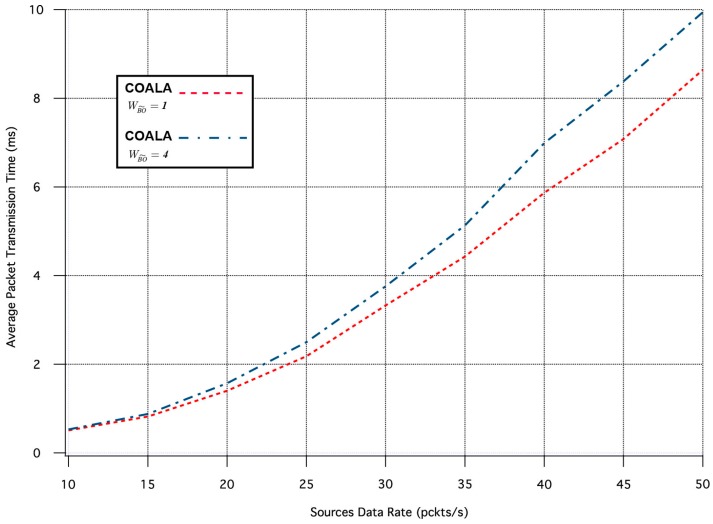
Average Packet Transmission Time vs. Traffic Load Rate for variable $\;w_{\tilde{BO}}.$

**Figure 10:**
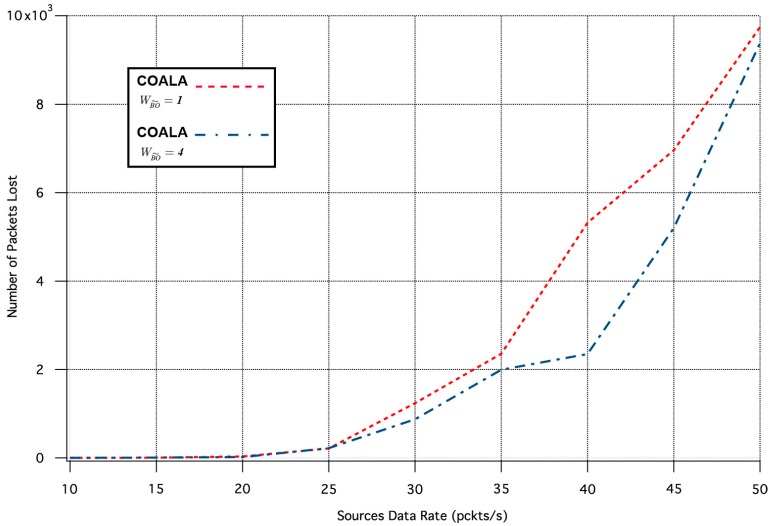
Number of Packets Lost vs. Traffic Load Rate for variable $\;w_{\tilde{BO}}$.

Similarly, the influence of the $\;w_{\tilde{BO}}$ variation in the two aforementioned metrics is depicted in Figure 9 and Figure 10 correspondingly.

The increase of $\;w_{\tilde{BO}}\;$ makes the buffer occupancy criterion have primary priority within the accumulated cost function. The examination of Figure 9 and Figure 10 confirms that this increase improves the reliability of the data transmission at the expense of the throughput. This is because data are preferred to be relayed through nodes that have less traffic load although this may involve longer paths.

## 5. Conclusions

In this research article, a novel lightweight scheme named COALA, which aims at preventing the diffusion of imminent congestion in WSNs through alternative path routing was introduced. The innovation of this proposed scheme lies in the fact that it takes into consideration a certain number of both invariant and variable factors that affect the probability of congestion occurrence along with other crucial factors like energy efficiency.

The efficacy of COALA protocol was evaluated through simulation tests in comparison with an advanced scheme of this kind, named DAlPaS Hard. The first comparative advantage of COALA is that it incorporates the use of the so-called vicinity index during the initial determination of routing paths, which makes nodes having a lot of possible data receivers and few data providers be favored. Therefore, traffic load may be distributed in a more balanced manner. Additionally, COALA introduces a multivariable cost function, which takes into consideration not only the metrics that DAlPaS Hard suggests, but also the popularity index, the vicinity index and the geographical distance of this node. Thus, congestion caused due to upper-level neighboring nodes that are repetitively busy, or have many probable data suppliers and few data recipients or are located at more distant locations, can be avoided. As a result, COALA achieves the reduction of transmission delays, lost packets rates, and energy dissipation.

Additionally, it was shown that the simple yet effective algorithm of COALA is able to accommodate to the performance criteria of each individual application through the appropriate adjustment of the weighting of its main cost function.

The authors of this article intend in future research work to enhance the herein-proposed protocol by either incorporating criteria based on well-known algorithms for energy efficiency [20,21,22], QoS [23] and security [24]. The convergence with game theoretic approaches [25], and the adaption of the proposed algorithm to standards for IPv6 routing in LLNs [26] are also under consideration.

## Figures and Tables

**Table 1:** Characteristic features of congestion avoidance and control protocols.

Protocol	Metric Considered	Congestion Notification	Congestion Mitigation/Avoidance
CODA [7]	Buffer occupancy and channel load	Explicit	Traffic Control
Ahmad and Turgut [8]	Buffer occupancy and characteristic ratio	Implicit	Traffic Control
PACA [9]	Buffer occupancy, characteristic ratio, distance, time of use	Implicit	Resource Control
DAlPaS [10]	Buffer occupancy, channel load, energy	Implicit	Resource Control
TALONet [11]	Buffer occupancy	Implicit	Traffic and Resource Control
HTAP [12]	Buffer occupancy, energy	Implicit	Resource Control
TADR [13]	Buffer occupancy	Implicit	Resource Control
ECODA [14]	Dual buffer threshold and weighted buffer difference	Implicit	Traffic Control
TARA [15]	Buffer occupancy and channel load	Explicit	Resource Control
PASCCC [16]	Buffer occupancy, type of content	Implicit	Traffic Control
COALA	Buffer occupancy, popularity index, energy, distance, vicinity index	Implicit	Resource Control

**Table 2:** Simulation Parameters.

Parameter	Value
Topology size	1000 m × 1000 m
Number of nodes	100
Number of different topologies	100
Number of simulation runs for the same topology	30
Node transmission range	50 m
Node sensing range	50 m
Node buffer size	10 packets
Initial node energy	1.5 J–2.5 J
Event range	100 m–400 m
Event range increase step	100 m
